# Unraveling the effects of sulfamethoxazole on the composition of gut microbiota and immune responses in *Stichopus variegatus*

**DOI:** 10.3389/fmicb.2022.1032873

**Published:** 2022-11-07

**Authors:** Chunming Tan, Wang Zhao, Weigeng Wen, Xu Chen, Zhenhua Ma, Gang Yu

**Affiliations:** ^1^Tropical Aquaculture Research and Development Center of South China Sea Fisheries Research Institute, Chinese Academy of Fishery Sciences, Sanya, China; ^2^Key Laboratory of South China Sea Fishery Resources Exploitation and Utilization, Ministry of Agriculture and Rural Affairs, Guangzhou, China; ^3^College of Food Science and Nutritional Engineering, China Agricultural University, Beijing, China; ^4^Sanya Tropical Fisheries Research Institute, Sanya, China

**Keywords:** *Stichopus variegatus*, antibiotic, high-throughput sequencing, intestinal microbiota, immune enzymes

## Abstract

The aim of this work was to reveal the changes in gut microbiota composition and immune responses of sea cucumber (*Stichopus variegatus*) after being affected by different doses of sulfamethoxazole. In this study, the bacterial 16S rRNA of gut microbiota were analyzed by high-throughput sequencing, and the activities of immune enzymes [lysozyme (LZM), phenoloxidase (PO), alkaline phosphatase (AKP), and acid phosphatase (ACP)] in the gut of *S. variegatus* were determined. The results showed that the gut microbiota presented a lower richness in the antibiotic treatment groups compared with the control group, and there were significant differences among the dominant bacteria of different concentration treatments. At the genus level, the abundance of *Escherichia*, *Exiguobacterium*, *Acinetobacter*, *Pseudomonas*, and *Thalassotalea* were significantly decreased in the 3 mg/L treatment group, while *Vibrio* was significantly increased. Furthermore, the 6 mg/L treatment group had less effect on these intestinal dominant bacteria, especially *Vibrio*. The changes in relative abundance of *Vibrio* at the species level indicated that lower concentrations of sulfamethoxazole could enhance the enrichment of *Vibrio mediterranei* and *Vibrio fortis* in *S. variegatus* more than higher concentrations of sulfamethoxazole. Meanwhile, the 3 mg/L treatment group significantly increased the activities of PO, AKP, and ACP, and decreased the activity of LZM. These results suggested that lower doses of sulfamethoxazole have a greater effect on the gut microbiota composition and immune responses in *S. variegatus* and may increase the risk of host infection.

## Introduction

*Stichopus variegatus* is an important aquaculture species in China with high nutrition and economic value (Wang J. J. et al., 2015). In addition to being edible, *S. variegatus* also has many important medicinal components, such as active saponins, acid mucopolysaccharides, and sulfated polysaccharides (Thinh et al., 2018; Kokadir et al., 2021). These biologically active compounds are good functional food ingredients. Consequently, the aquaculture and consumption of *S. varigatus* is increasing. In recent years, with the expansion of intensive aquiculture of *S. variegatus*, the breeding industry began to frequently explode various bacterial diseases, and the dispensable and inexpensive method to deal with pathogenic infections is still to use a certain amount of antibiotics.

Sulfamethoxazole is a widespread antibiotic, because of their physicochemical properties and broad spectrum of against antibacterial activity, has been used in aquaculture to control the occurrence of aquatic animal diseases (Payán et al., 2011; Zhang et al., 2015). In China, there is a severe situation that sulfamethoxazole misuse or abuse is widespread, because of the weak public consciousness and lack of reasonable supervision, especially in the breeding of sea cucumbers. However, sulfamethoxazole has the characteristics of low bioavailability and high water solubility. The large amount of unabsorbed antibiotics not only poses a serious ecological threat to the environment, but also severely disturbs the intestinal homeostasis of organisms and leads to the emergence of antibiotic-resistant strains (Li et al., 2015; Zhao et al., 2019b). Studies showed that although antibiotics control the occurrence of diseases to some extent, it also affects the structure of animal intestinal microflora, studies on the changes of intestinal microflora in aquaculture animals caused by antibiotics also attracted attention (Stanton, 2013; Wang R. X. et al., 2015; Zhao et al., 2019b). Gut microbiota are important for host growth and survival, they can help the host facilitate digestion, maintain energy balance, prevent pathogen colonization, and improve immunity (Ming et al., 2020). It plays crucial roles in boosting the immune-related activities and degrading toxic compounds in the host (Tremaroli and Backhed, 2012; Purchiaroni et al., 2013; Zhao et al., 2016). Especially bacterial pathogens (e.g., *Escherichia*, *Shigella*, and *Vibrio*, etc.), of which *Vibrio* species are the main opportunistic bacterial pathogens responsible for diseases in marine animals (Kang et al., 2014). However, systematic analysis of the effects of antibiotics on the gut microbiota and immune responses in *S. variegatus* has not been performed.

The gut microbiota of aquatic animals is an integral part of intestinal tract, it is interdependent and mutually restrictive with the host and the aquatic environment in which it is resided (Wang et al., 2010). The homeostatic balance of the gut microbiota in sea cucumber is sensitive to these factors and causing diseases like rot skin syndrome, mycosis, and bacterial ulcer disease (Zhao et al., 2016; Zhang et al., 2018). Previously, the study on the gut microbiota of sea cucumber is mainly through the analysis of culturable microorganisms, however, 85–99% of bacteria in nature cannot be isolated and cultured (Zhang et al., 2011). Comprehensive information on animal gut microbiome failed to truly reflect, as only a small fraction of the microbes was isolated and characterized (Iacumin et al., 2009). Although the combination of 16S rRNA and genetic fingerprinting technologies can be used to identify the dominant microflora in the aquatic animal intestine (Shokralla et al., 2012), it is vulnerable to the effects of the gene copy number.

High-throughput sequencing was regarded as a new technology in microbiota study, which has been successfully used in many disciplines to analyze the complex bacterial ecosystem of the gut, such as phytoplankton, marine and intestinal environment, fermented food, and human health and disease (Qiao et al., 2019; Ming et al., 2020; Chen Y. et al., 2022). These new analytical approaches have significantly accelerated the characterization of animal bacteria in several phenotypes and diseases by metagenomic and 16S rRNA-based techniques (Zoetendal et al., 2008; Fre-Mont et al., 2013; Zhao et al., 2016). It provides a high number of reads at a relatively low cost, and thousands of bacteria can be identified in each sample by sequence analysis. Sequencing-based molecular techniques can provide a comprehensive dissect of the dynamics of gut microbiota and reveal the association between changes in microbiota composition and disease (Yu et al., 2013; Shang et al., 2018; Zhuang et al., 2019). In this study, a high-throughput sequencing approach was used to reveal the characteristics of *S. variegatus* gut microbiota after being affected by sulfamethoxazole. And the immune-related enzyme activities of *S. variegatus* were also analyzed. We aimed to investigate the advantages and disadvantages of sulfamethoxazole in aquaculture, and then provide a reference for the administration of sulfamethoxazole in the aquaculture industry.

## Materials and methods

### Sample collection

The *S. variegatus* were caught from an aquaculture base in West Island of Sanya in April 2019, bodyweight 152.17 ± 26.89 g. We randomly divided 90 sea cucumbers into three pools (500 L each, 30 sea cucumber per pool), and then allowed the sea cucumbers to acclimate for a week in the culture pool of the Tropical Aquaculture Research and Development Center. Feeding with artificial feed (composed of Sargassum powder, Sargassum thunbergii powder, sea mud, sand, and sea cucumber bait in equal proportion). After the acclimation period, the experiment started and continued for 12 days. During the experiment, sea cucumbers were fed artificial feed once daily at 16:00. The water temperature, salinity, and pH of the seawater during the period were (25 ± 0.5)°C, 32 ± 0.8, and 7.8 ± 0.2, respectively. The control group (HCG) was treated with no added sulfamethoxazole, the HATG treatment group were supplemented with sulfamethoxazole (3 mg/L water volume), while the HHC treatment group were supplemented with sulfamethoxazole (6 mg/L water volume). The complete intestine samples of *S. variegatus* were collected after the end of experimentation in a pool with sulfamethoxazole. Each group were performed in three replicates, and all samples were immediately stored at −80°C and processed within a month.

### Microbial community structure analysis

Total genomic DNA of the samples was extracted using Stool DNA Kit (Refer to QC report for specific kit) according to manufacturer’s protocols. The V3 and V4 regions of 16S rRNA were amplified by specific primer combination (forward primers: 5′-CCTACGGRRBGCASCAGKVRVGAAT-3′ and reverse primers: 5′-GGACTACNVG GGTWTCTAATCC-3′). At the same time, indexed adapters were added to the ends of the 16S rRNA amplicons to generate indexed libraries ready for downstream NGS sequencing on Illumina Miseq. PCR reactions were carried out in 25 μl mixture containing 2.5 μl TransStart Buffer, 2 μl dNTPs, 1 μl each primer, and 20 ng template DNA. The PCR products were checked by 2% agarose gel electrophoresis and quantified the library to 10 nM using a Qubit fluorescence quantifier. The amplicon libraries were constructed using the TruSeq® DNA Library Prep Kit (Illumina Inc., San Diego, California, United States), and sequencing was performed on an Illumina MiSeq PE300 platform (Illumina, San Diego, CA, United States). The raw sequence datasets of samples obtained in this study have been uploaded to the NCBI Sequence Read Archive (SRA), BioProject PRJNA874578.

Quality filtering on merged sequences was performed by QIIME (version 9.1), and the sequences were compared with the reference database (RDP Gold database) using UCHIME algorithm to detect chimeric sequence. Effective sequences were clustered into operational taxonomic units (OTUs) using the clustering program VSEARCH (1.9.6) against the Silva 132 database pre-clustered at 97% sequence identity. The OTUs were taxonomically identified using the Ribosomal Database Program (RDP) classifier. The RDP classifier uses the Silva 132 database, which has taxonomic categories predicted to the genus level (Quast et al., 2013; Yilmaz et al., 2014). The OTU cluster information, rarefaction curves, and alpha diversity indexes (Shannon and Simpson index, Chao1 richness index, and Good’s coverage) were calculated by QIIME program (Kang et al., 2021). The beta diversity was carried out using UniFrac analysis (Hamady et al., 2010) and principal coordinates analysis (PCoA), the ANOSIM was used to perform a hypothesis test of the microbiome (Kim et al., 2021). The significant differences in community composition between groups based on community abundance data at genus level were analyzed by Metastats analysis. The Kruskal-Wallis test was used to detect taxa that exhibit differences in abundance between the two groups of microbial communities, and the multiple hypotheses testing of rare frequency data and false discovery rate (FDR) analysis were used to assess the significance of the observed differences.

### Immune indexes

The activity of immune enzymes in the samples were tested with corresponding kits (Beijing Solarbio Science & Technology Co., Ltd., Beijing, China), including lysozyme (LZM, No. A050-1), phenoloxidase (PO, No. H247), alkaline phosphatase (AKP, No. A059-2), and acid phosphatase (ACP, No. A060-2). The activity of LZM was determined according to the fact that LZM can lyse bacteria and enhance the light transmittance of turbid bacterial liquid, and the results were expressed as U per mg protein. The protein concentration of the samples was tested using a BCA kit (No. A045-3).

The content of PO was determined according to the kit instructions. It is based on the competition of enzymes and antibodies against solid phase antigens, followed by the formation of immune complexes. The results were expressed as mg per g protein.

Alkaline phosphatase and ACP can catalyze the formation of free phenol from disodium phenyl phosphate under acidic and basic conditions, respectively. The phenol reacts with 4-aminoantipyrine in alkaline solution and is oxidized by potassium ferricyanide to form red quinone derivatives, its enzymatic activity can be calculated based on the absorbance value at 510 nm (Wei et al., 2022). One unit of enzymatic activity is defined as the catalytic production of 1 μM phenol per milligram of protein per minute at 37°C, and the results were expressed as U per mg protein.

### Statistical analysis

The data were processed using Excel 2016 and expressed as mean ± SD. The significant differences among samples were analyzed through SPSS v21.0 (SPSS Inc., Chicago, IL, United States). A one-way ANOVA and Duncan multiple comparison test were used to compare the effect of sulfamethoxazole on the differences for LZM, PO, AKP, and ACP. The *p* < 0.05 represented has a significant difference.

## Results

### Characteristics of the sequencing results and alpha diversity

**Figure 1:**
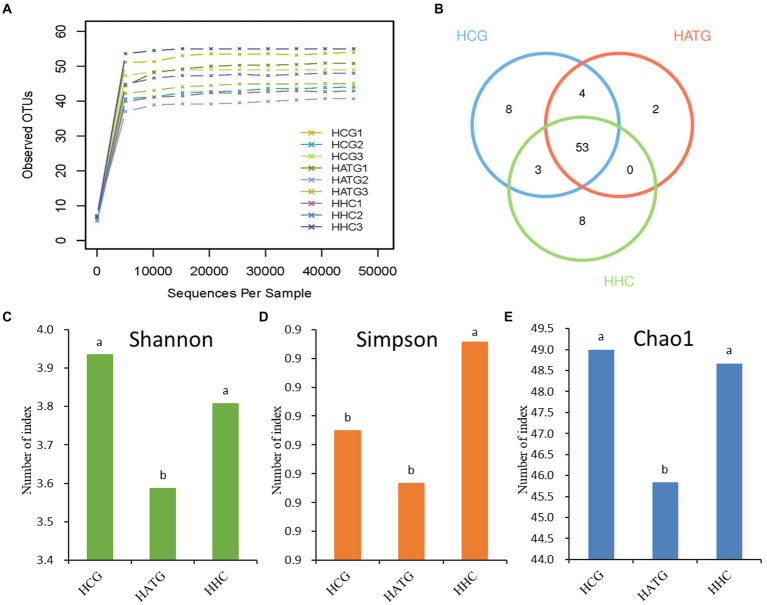
The Rarefaction Curve **(A)**, Venn diagram **(B)**, Shannon diversity index **(C)**, Simpson diversity index **(D)**, and Chao1 richness index **(E)** for various samples. The HCG, HATG, and HHC represent control group, 3 and 6 mg/L sulfamethoxazole treatment group, respectively. Different letters obtained by one-way ANOVA followed by Duncan’s test indicated significant differences at *p* < 0.05.

A total of 664,189 high quality reads of 16S rRNA sequences were obtained from the nine samples with 73,799 sequence reads per sample, the average length of sequence reads was 459.75 bp, and the coverage reached 99.9% (Table 1). The rarefaction curves showed a similar pattern of reaching plateau and the final trend is saturated (Figure 1A). These results showed almost all the OTUs in the datasets have already been captured.

**Table 1:** Sample information and sequence statistics.

Sample ID	Sequences number	Average length (bp)	OTUs	Good’s coverage
HCG1	78,879	461.20	54	0.999
HCG2	80,842	462.32	44	0.999
HCG3	72,524	459.01	49	0.999
Average	77,415	460.84	49	0.999
HATG1	71,176	459.65	51	0.999
HATG2	70,939	459.08	41	0.999
HATG3	59,607	455.64	45	0.999
Average	67,240	458.12	45.7	0.999
HHC1	79,968	461.34	43	0.999
HHC2	65,468	448.54	48	0.999
HHC3	84,786	460.41	55	0.999
Average	76,740	456.76	48.7	0.999

The Venn diagram could reflect the difference among HCG, HATG, and HHC groups. There were 68, 59, and 64 OTUs in HCG, HATG, and HHC, and the ratio of their common OTUs were 77.9, 89.8, and 82.8%, respectively (Figure 1B) It indicated that the sulfamethoxazole significantly changed the microbial composition in *S. variegatus* intestine.

The community diversity can generally be reflected by Shannon index and Simpson index, and the greater the Shannon value or the lower the Simpson value, the higher the species diversity. According to the results, two indices hinted that the control group (HCG) has the highest community diversity (Figures 1C,D). In addition, Chao proposed that the index of the number of OTUs in the sample estimated by the Chao1 algorithm can be well used for the estimation of species evaluation in ecology (Pitta et al., 2010). Chao1 index represent the community richness, the greater the Chao1 index, and the higher the community richness. As shown in Figure 1E, the highest Chao1 index is control group, while the lowest Chao1 index is HATG group, which suggest that the community richness of the gut microbiota of *S. variegatus* decreased after treated by sulfamethoxazole, and the low doses of sulfamethoxazole had a greater impact.

### Analysis of beta diversity

**Figure 2:**
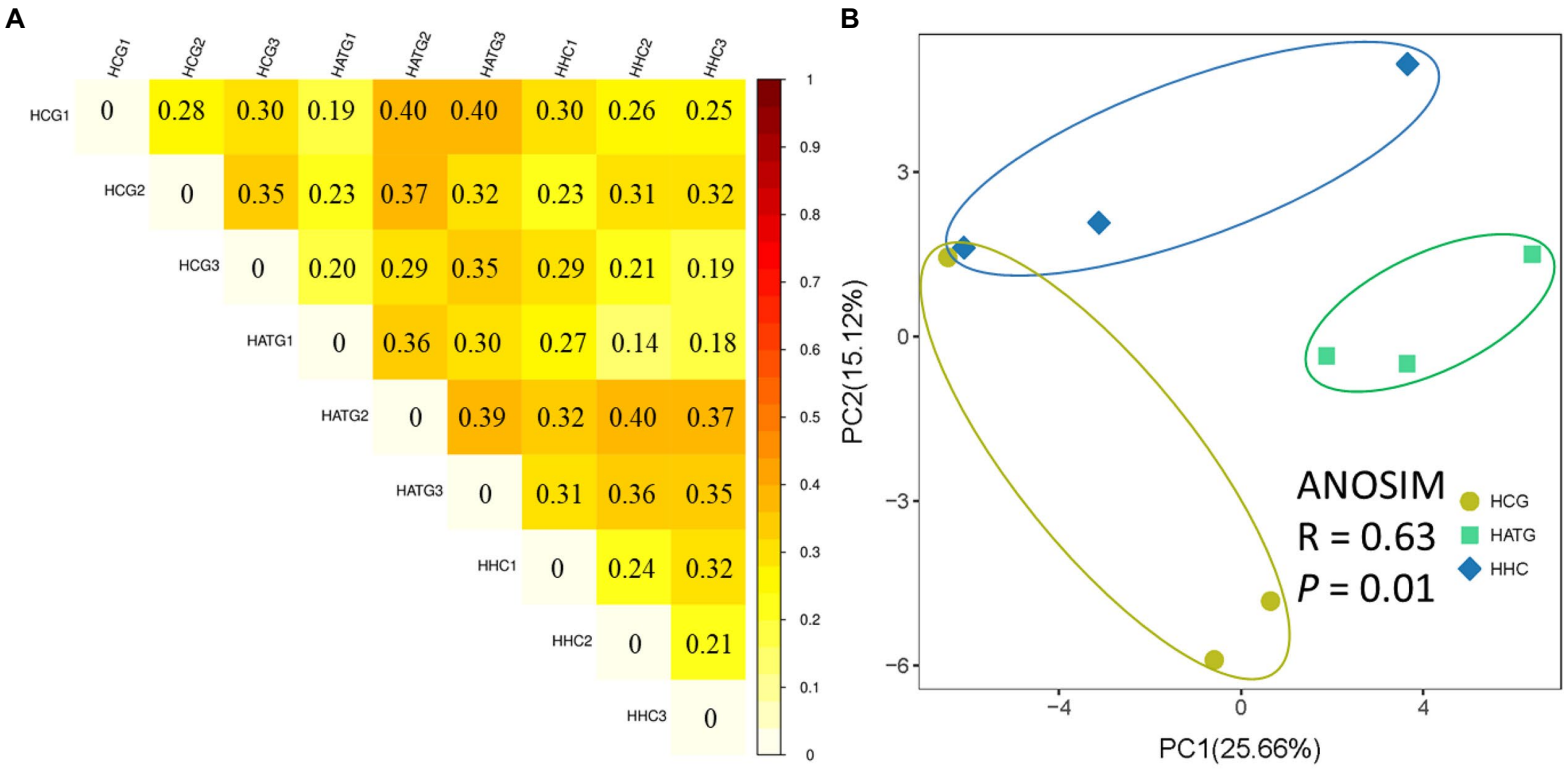
Unweighted UniFrac difference analysis of the diversity among samples **(A)** and principal co-ordinates analysis (PCoA) of bacterial community of *Stichopus variegatus* **(B)**.

The differences in microbiota between samples can be compared through calculating the distance between samples by using the richness and evolutionary information of sample sequences (Lozupone and Knight, 2012). In this study, the UniFrac analysis was used to calculate the distance matrix of samples, and the differences were reflected in a heat-map with color changes. The defined light and dark colors in the heat-map represent the degree of difference between the two samples. Compared with control group, the HATG treatment group had a higher difference coefficient in genus diversity (Figure 2A). This result indicated that the effect of HATG treatment group on the diversity of the gut microbiota of *S. variegatus* was greater than that of HHC group.

The PCoA analysis based on the relative abundance of OTUs showed that the differences in microbial community composition was significant (*p* < 0.05; Figure 2B). The HCG samples were clearly separated from the HATG and HHC samples, suggesting that the microbial community composition changed greatly and the effect of sulfamethoxazole on the microbial community composition was significant and greater at the low doses of sulfamethoxazole.

### Changes in community abundance and composition

**Figure 3:**
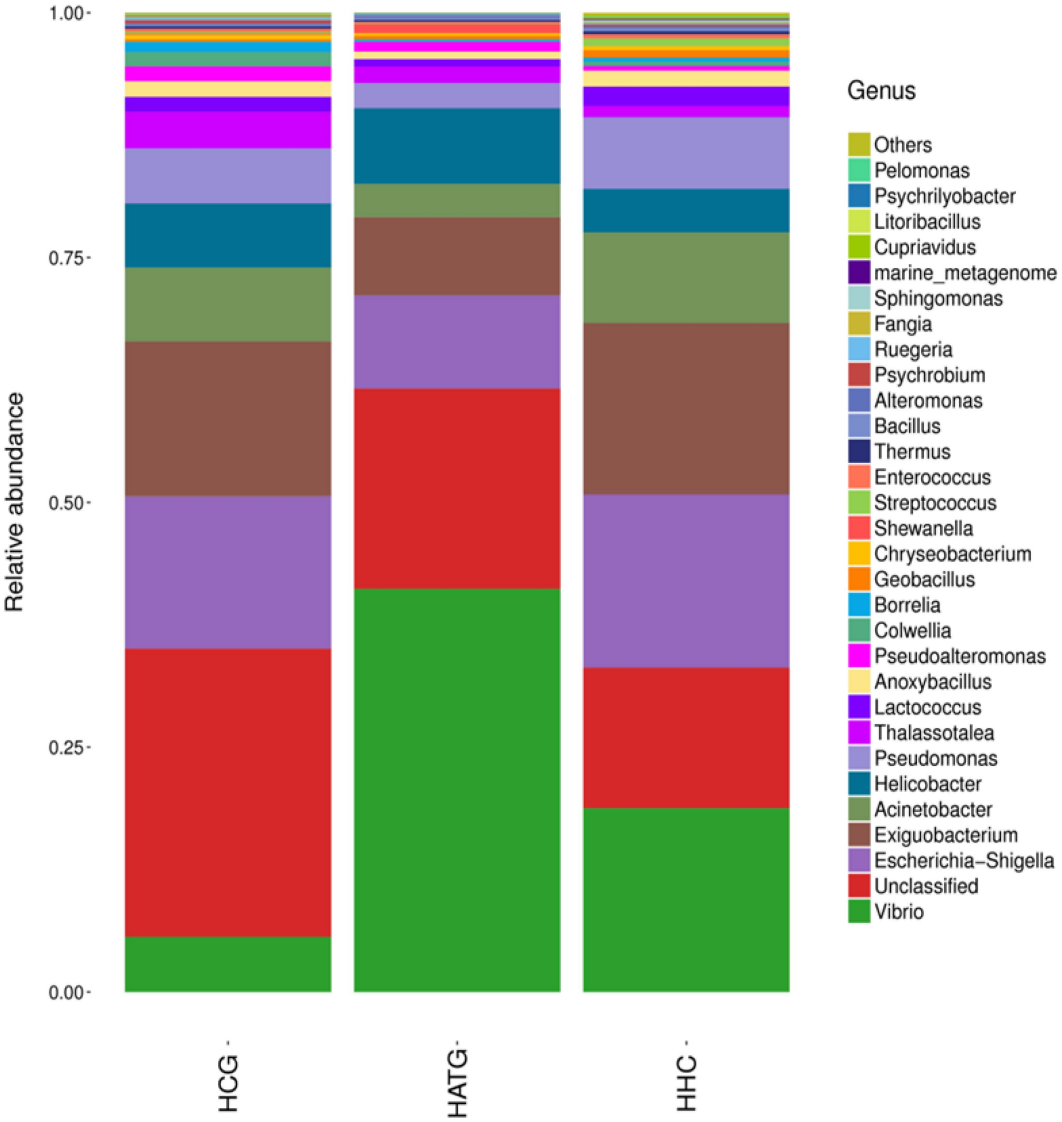
Composition and relative abundance of bacterial communities in genera of the different treatment groups.

In this study, statistical analysis was used to observe the community structure of samples at different taxonomic levels. At the genus level, data for the top 30 microbial populations were analyzed. As shown in Figure 3, *Vibrio*, *Escherichia*-*Shigella*, *Exiguobacterium*, *Acinetobacter*, *Helicobacter*, and *Pseudomonas* constituted six common dominant genera in HCG, HATG, and HHC groups, which accounted for 56.74, 72.37, and 74.97% of the total sequencing number. However, *Thalassotalea*, *Lactococcus*, *Anoxybacillus*, and *Pseudoalteromonas* were also the dominant genera in the HCG group. Compared with control group, the abundant of *Escherichia*-*Shigella*, *Exiguobacterium*, *Acinetobacter*, *Pseudomonas Thalassotalea*, *Lactococcus*, *Anoxybacillus*, and *Pseudoalteromonas* were significantly decreased in the HATG groups, while *Vibrio* increased significantly. And the ratio of *Vibrio* in HATG group was much higher than that in the control group and HHC group, indicating that the *Vibrio* in gut microbiota of *S. variegatus* was more sensitive to the effects of lower concentrations of sulfamethoxazole (Figure 3).

### Analysis of significant differences in community structure between groups

**Figure 4:**
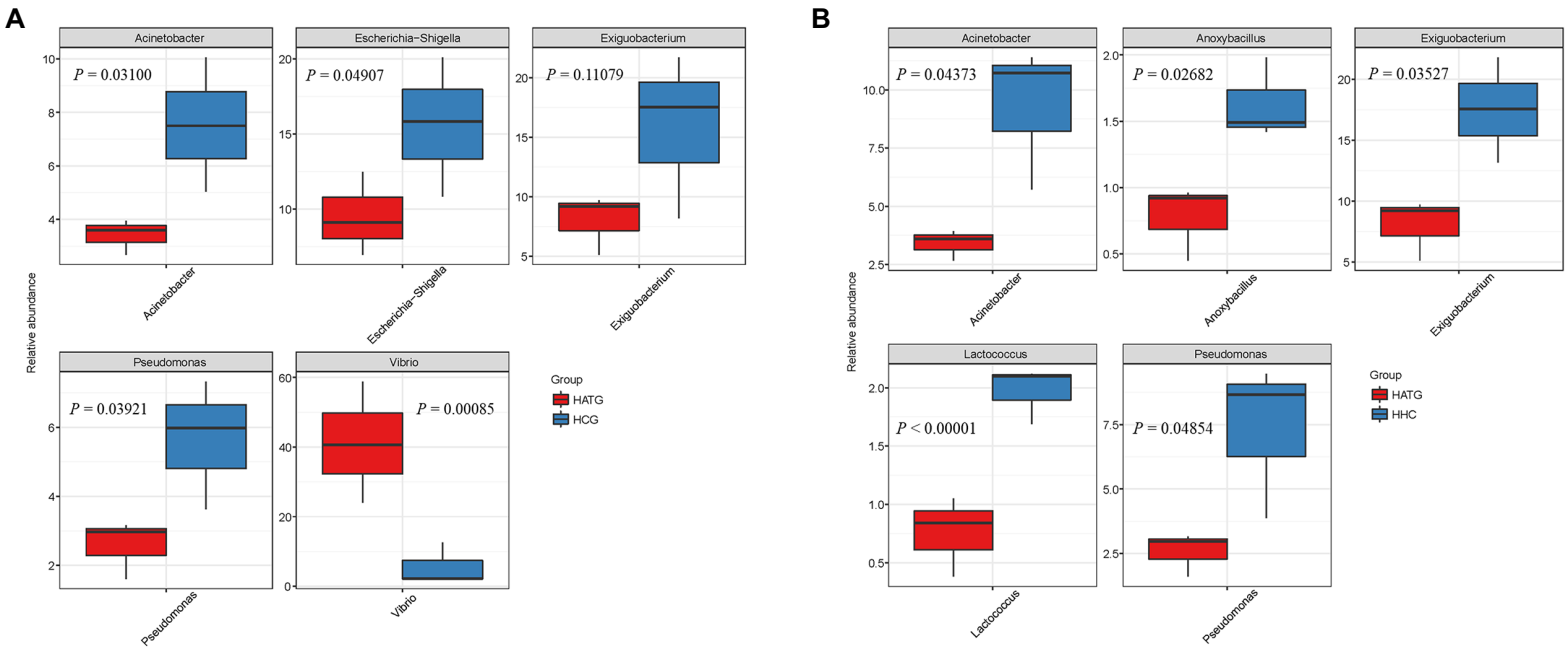
Comparison of relative abundance differences of the bacterial communities between groups, **(A)** group HCG and HATG, **(B)** group HATG and HHC. A Kruskal-Wallis test was used to detect the differences in abundance between the two groups of microbial communities.

Based on community abundance of different groups, the five top genera significant differences between groups were compared. A Kruskal-Wallis test was used to detect the classification of abundance differences between two groups of microbial communities, and multiple hypothesis test and false detection rate (FDR) analysis of rare frequency data are carried out. As shown in Figure 4A, the *Acinetobacter*, *Escherichia*-*Shigella*, *Exiguobacterium*, *Pseudomonas*, and *Vibrio* were most significantly different (*p* < 0.05) between HCG and HATG. Among these five genera, *Vibrio* was significantly more abundant in HATG group compared with HCG group, while the opposite was observed for other genera. Compared with HATG group, the HHC showed a significantly higher bacterial community abundant in *Acinetobacter*, *Anoxybacillus*, *Exiguobacterium*, *Lactococcus*, and *Pseudomonas* (Figure 4B). The results indicated that lower concentration sulfamethoxazole was more effective in reducing the abundance of gut microbiota of *S. variegatus*, but greatly increased the abundance of *Vibrio*. This means that lower concentration sulfamethoxazole can play a better antibacterial effect, but increase the risk of *Vibrio* infection in *S. variegatus*.

### Relative abundance changes of *Vibrio* species in *Stichopus variegatus*

**Figure 5:**
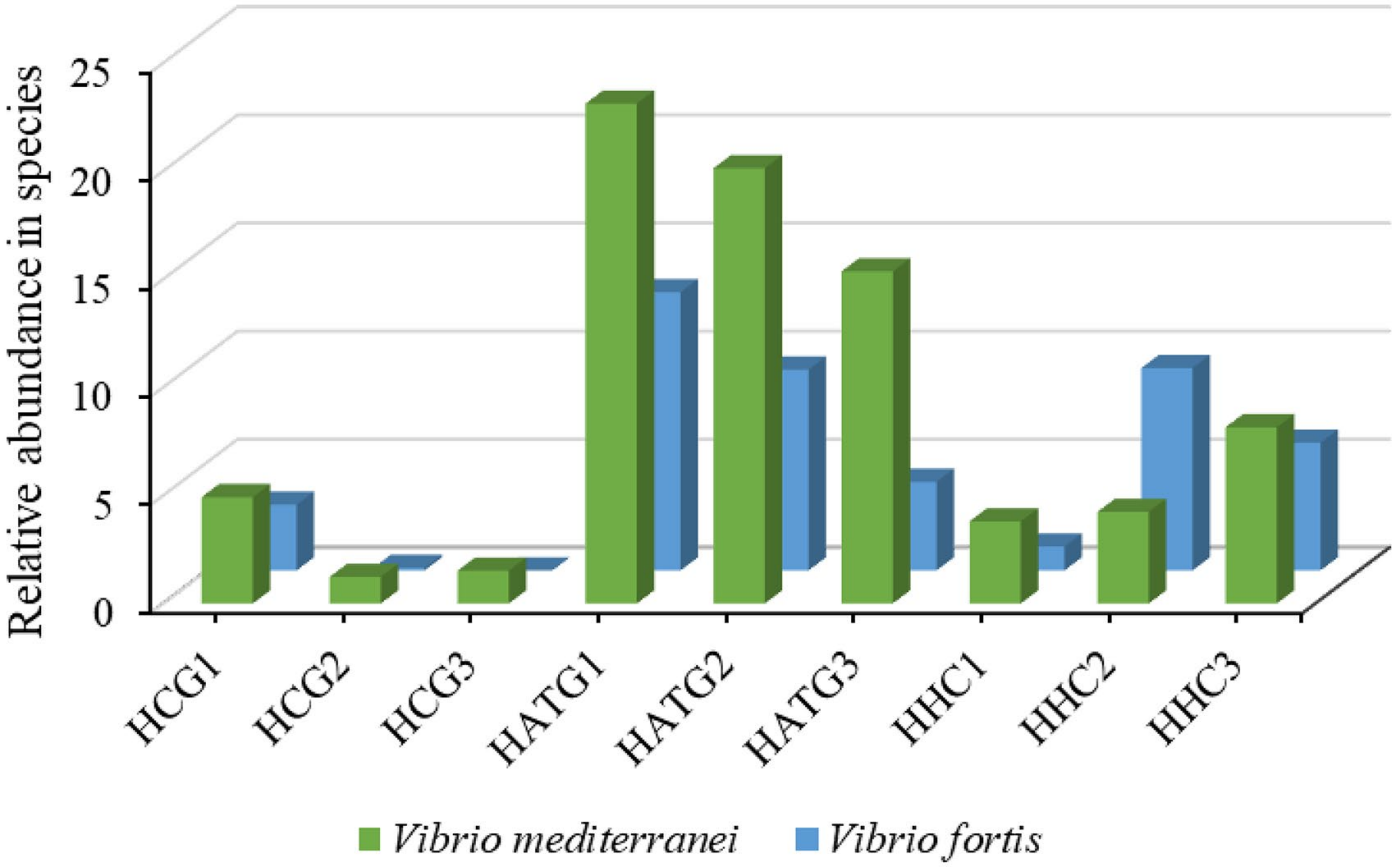
The relative abundance changes of *Vibrio* species of Intestinal microbiota in *Stichopus variegatus*.

Furthermore, the relative abundance of *Vibrio* at the species level was analyzed, as shown in Figure 5. In this study, two types of *Vibrio* species were identified, namely *Vibrio mediterranei* and *Vibrio fortis*. Compared to the group not treated with sulfamethoxazole (HCG), the abundance of *V. mediterranei* was significantly increased after sulfamethoxazole treatment at 3 mg/L (HATG). However, the 6 mg/L sulfamethoxazole treatment group (HHC) had a lower effect on *V. mediterranei*. And the effect of sulfamethoxazole on *Vibrio fortis* in the gut of *S. variegatus* was consistent with the trend of *V. mediterranei*. The results further indicated that lower concentrations of sulfamethoxazole could enhance the enrichment of *Vibrio* in *S. variegatus* more than higher concentrations of sulfamethoxazole.

### Effects on the activities of immune-related enzymes in *Stichopus variegatus*

**Figure 6:**
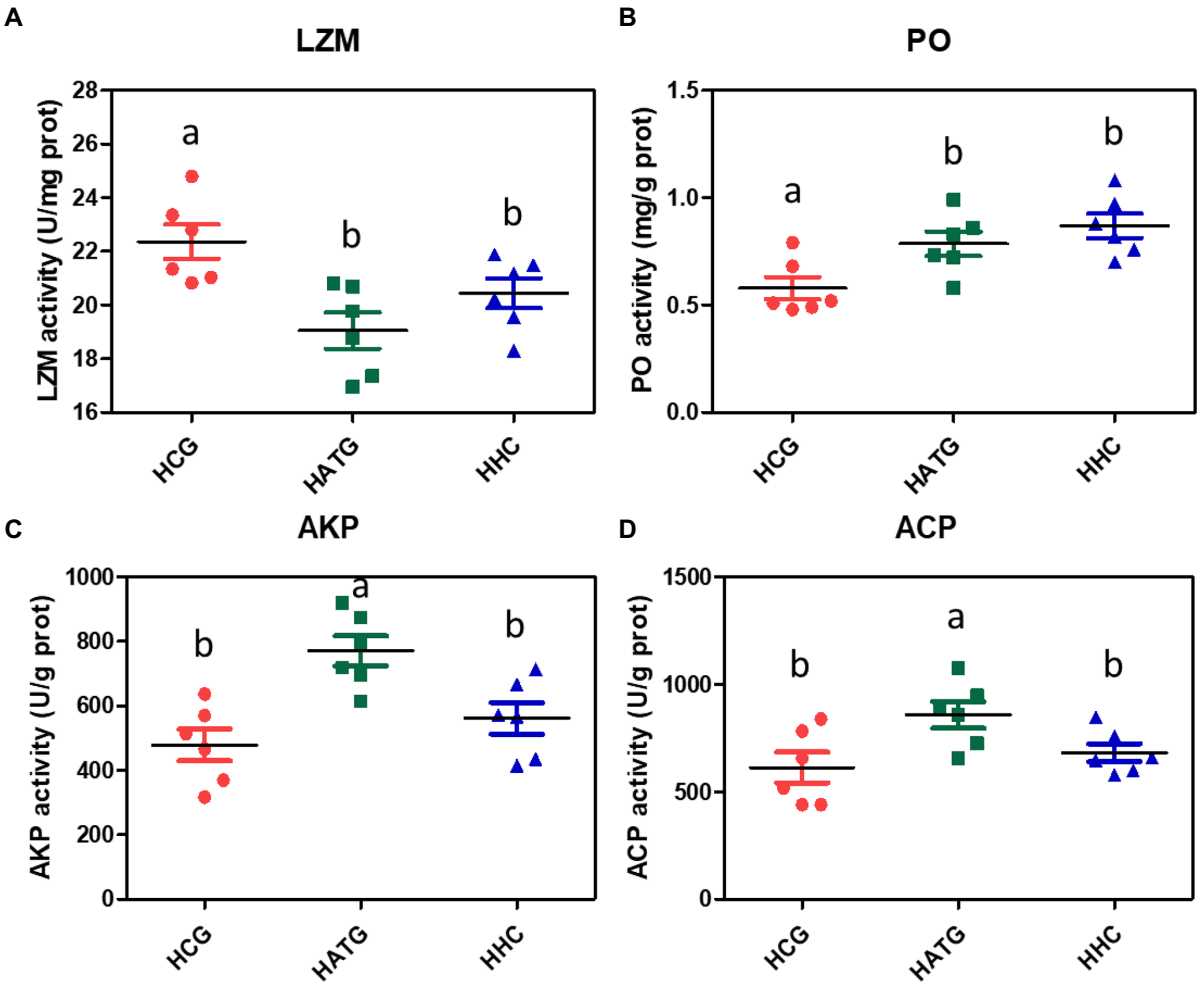
Changes in the activities of immune-related enzymes in *Stichopus variegatus* after treated by sulfamethoxazole. **(A)** lysozyme activity (LZM), **(B)** phenoloxidase activity (PO), **(C)** alkaline phosphatase activity (AKP), and **(D)** acid phosphatase (ACP). Data were expressed as mean ± SD (*n* = 6). The different superscript letters indicated significant differences from other groups (*p* < 0.05).

The LZM activity of *S. variegatus* was significantly changed after being affected by sulfamethoxazole (*p* < 0.05), as shown in Figure 6A. The activity of LZM decreased in both HATG and HHC treatment groups, and the decrease was more pronounced in the HATG treatment group. The changes in PO activity after the sulfamethoxazole exposure showed a trend of increasing with the concentration of sulfamethoxazole (Figure 6B). However, there was no significant difference between the HATG and HHC treatment groups (*p* > 0.05). The alkaline phosphatase and acid phosphatase activities in *S. variegatus* were also affected by sulfamethoxazole exposure, and they showed a similar trend. The lower concentration antibiotic treatment group significantly increased the activities of ACP and AKP, while there was no significant difference between the higher concentration antibiotic treatment group and the control group (Figures 6C,D).

### Correlation analysis between the bacterial abundance and immune enzymes

**Figure 7:**
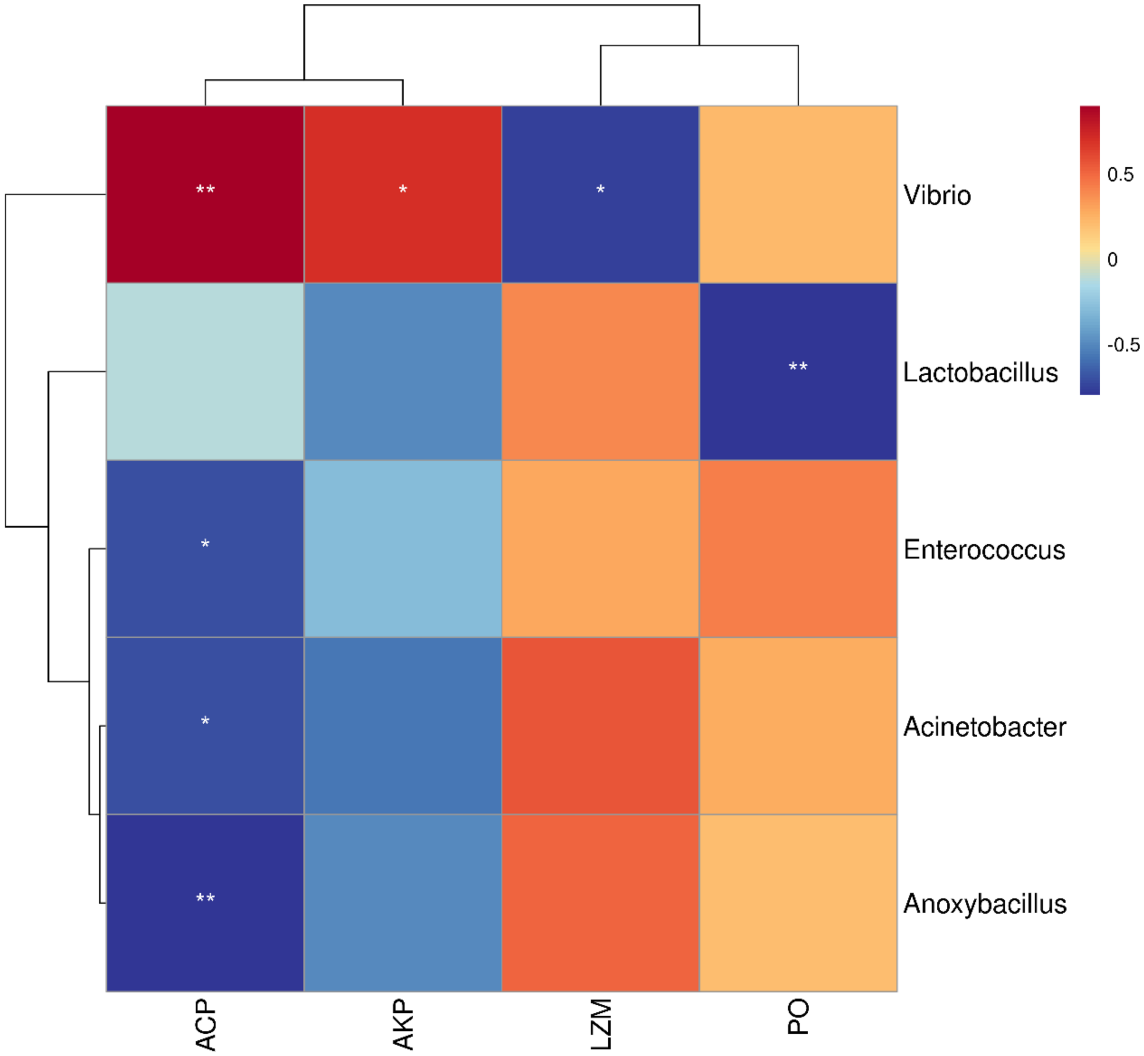
Analysis of the correlation between microbiota and immune enzymes. Microbiota was considered at the OTU level and statistically significant Spearman correlations were calculated among samples. ^*^ represented *p* < 0.05, ^**^ represented *p* < 0.01.

The correlation between microbiota and immune-related enzymes of *S. variegatus* was analyzed by Spearman correlation analysis (Figure 7). Dominant bacteria *Vibrio*, *Anoxybacillus*, *Acinetobacter*, *Lactobacillus*, and *Enterococcus* showed a significant correlation with the immune enzymes (*p* < 0.05). Among them, *Vibrio* had the highest abundance at the HATG group, which positively correlated with activities of ACP, AKP, and PO, and negatively correlated with LZM. While *Lactobacillus* showed a positive correlation with the activities of LZM, and negatively correlated with PO and AKP. Besides, *Enterococcus*, *Acinetobacter*, and *Anoxybacillus* showed a similar correlation with immune enzymes, which is positive correlation with the activities of LZM and PO, and negatively correlated with ACP and AKP.

## Discussion

Accumulating scientific research-based evidence is driving our increased awareness of the significance of the antibiotics to the healthy and homeostatic operation of the aquatic animals (Wang J. J. et al., 2015; Manage, 2018). Antibiotics are gradually attracting global attention as a new type of pollutant. To investigate the effects of sulfamethoxazole on aquatic animals might provide a reference for the administration of sulfamethoxazole in aquaculture. In this work, high-throughput sequencing technique was applied to reveal the gut microbiome characteristics of *S. variegatus* after being affected by sulfamethoxazole, and a comprehensive analysis of the sequence abundance and diversity of the gut microbiota of the sulfamethoxazole treated and non-sulfamethoxazole treated groups were performed. We found that OTUs and some bacterial genera varied among different treatments, indicating the abundance and diversity of bacterial communities in samples were changed by different concentrations of sulfamethoxazole. Angelakis et al. (2013) reported that the effects of antibiotics on changes in gut microbiota and functional alterations in animals depend on the type of antibiotic, dose, and duration of exposure.

The community richness and diversity of the gut microbiota in sulfamethoxazole treatment groups decreased compared with control group. The decrease of diversity will break the dynamic balance of intestinal microecology and cause instability of community structure (Zhao et al., 2018). Liu et al. (2011) reported that there is a relative dynamic equilibrium between the bacteria in the intestine, or between the animals and the intestinal bacteria, this state is of great significance to the maintenance of the host’s normal physiological function. The imbalance of gut microbiota of the sea cucumber is easy to cause disease such as inflammatory bowel disease, and this change in the structure of the gut microbiota may also be one of the causes of frequent disease in spring and autumn (Zhang et al., 2006; Zhao et al., 2019a). In this study, the effect of 3 mg/L sulfamethoxazole treatment groups on gut microbiota is greater than in 6 mg/L sulfamethoxazole exposed group. This may be a result of varying degrees of suppression of the normal microflora in the intestine that are sensitive to the drugs, while insensitive strains multiply under the action of higher concentration of antibiotics and induce other bacteria to produce resistance. Previous studies have demonstrated that antibiotic resistance can be rapidly induced and spread among bacterial species, resulting in harmful effects on aquatic ecology and aquatic organisms (Wang and Schaffner, 2011). The report of Butts et al. (2019) on establishment of antibiotic-induced dysbacteriosis model also confirmed that low concentration antibiotics induce dysbacteriosis in a method that is superior to higher concentration of antibiotics. This might be low-dose antibiotics have a greater impact on their metabolic capacity and the spread of antibiotic-resistant bacterial strains (Duan et al., 2020). Besides, the enterotype, genotypes, and different antibiotic sensitivities of individuals may also lead to low-dose antibiotic treatment induces dysbiosis by decreasing the diversity of the microbiome.

Our results indicated that the composition of gut microbiota of *S. variegatus* in phylum level are consistent with previous studies that *Proteobacteria* and *Firmicutes* have high abundance (Pagán-Jiménez et al., 2019; Zhao et al., 2019a; Chen J. et al., 2022). At the genus level, the results of heat-map showed that the community richness of the gut microbiota of *S. variegatus* decreased after treated by sulfamethoxazole, and *Vibrio*, *Escherichia*, *Exiguobacterium*, *Acinetobacter*, *Helicobacter*, and *Pseudomonas* were the dominant bacteria have the highest richness at the genus level. As for *Vibrio*, *Escherichia*, and *Acinetobacter* that played important roles in aquaculture, which were noticeably abundant in the naturally occurring animals (Hakim et al., 2015). However, *Vibrio* and *Escherichia* were considered intestinal pathogens that can cause diseases in aquatic animals (Ming et al., 2020). The *Vibrio* in low-concentration sulfamethoxazole treatment group showed the greatest richness, and the proportion of *Vibrio* species after treated by sulfamethoxazole has increased greatly. This result is probably due to the fact that *Vibrio* species are the main opportunistic bacterial pathogens responsible for diseases in marine animals (Kang et al., 2014). However, the significantly decreased abundance of *Escherichia* and *Acinetobacter* may be due to their sensitivity to sulfamethoxazole. Fu et al. (2008) reported that *Escherichia* sensitive to sulfonamides, streptomycin, chloramphenicol, etc.

After analyzing differences in relative abundance, we observed that the genera between the two groups were significantly different, which suggested that sulfamethoxazole influenced the composition of gut microbiota, and the effect varies with the concentration. In the study, the effect of low concentration of sulfamethoxazole treatment on gut microbiota is much greater than higher concentration. The result was consistent with the characteristics of gastrointestinal microbiota in mice that induced by an antibiotic (Butts et al., 2019). The sulfamethoxazole significantly increased the ratio of *Vibrio* and decreased the proportion of *Exiguobacterium*, *Acinetobacter*, and *Pseudomonas*. Wang R. X. et al. (2015) showed that many *Vibrio* strains have high resistant rates to antibiotics. In China, marine *Vibrio* species are the predominant multidrug-resistant bacteria in the mariculture environments studied, which have long been recognized as important reservoirs and vehicles of antimicrobial resistance (Thompson et al., 2005; Dang et al., 2009). Thompson et al. (2004) showed that *Vibrio* species have the ability to readily develop and acquire antimicrobial resistance in response to selective pressure, as well as their ability to spread resistance through horizontal exchange of genetic material. And this suggests that *Vibrio*, *Acinetobacter*, *Pseudomonas*, and *Exiguobacterium* may play an important role in balancing gut microbiota of *S. variegatus*. However, this is the first report of an abundance of *Pseudomonas* in low concentration of sulfamethoxazole treatment groups and the *Exiguobacterium* in high concentration treatment groups, more research must be conducted.

According to Liu et al. (2020), the antibiotics exposure might evoke immune responses in healthy aquatic organisms. In this study, the LZM activity of *S. variegatus* was significantly inhibited after exposure to sulfamethoxazole environment. This might be sulfamethoxazole suppresses the ability of *S. variegatus* to resist pathogen. Strzepa and Szczepanik (2013) reported that the composition of the gut microbiota affects the immune response. Through correlation analysis between the bacterial abundance and immune enzymes we found that the dominant gut microbiota *Vibrio*, *Anoxybacillus*, *Acinetobacter*, *Lactobacillus*, and *Enterococcus* in *S. variegatus* showed a significant correlation with the immune enzymes (*p* < 0.05). Especially the *Vibrio*, which showed a strong correlation with the activities of ACP, AKP, and LZM. This is the reason why ACP, AKP, and LZM enzyme activities were significantly higher or lower in the HATG-treated groups. Therefore, the change of immune enzymes of *S. variegatus* related to the influence of sulfamethoxazole on the community structure of its gut microbiota. However, the treatment of sulfamethoxazole significantly increased the PO activity of *S. variegatus*, although there was no significant difference between HATG and HHC. This may be related to the increased proportion of *Vibrio* in the sea cucumber gut after sulfamethoxazole treatment, because phenoloxidase plays an important role in inhibiting or even killing pathogenic microorganisms (Liu et al., 2007). In addition, AKP and ACP activities showed a consistent pattern of changes, that is, treatment with lower concentrations of sulfamethoxazole significantly increased their activities. The results suggested that lower concentration sulfamethoxazole might make *S. variegatus* easier to against pathogens and foreign bodies. These results may be an important piece of evidence in the fact that *S. variegatus* is more susceptible to diseases in artificial aquaculture systems than in natural growth, but this needs to be confirmed further. In addition, it is necessary to conduct more research to characterize how antibiotic differences directly affect the bacterial populations present in intestinal samples.

## Conclusion

The structure of the gut microbiota in *S. variegatus* changed dramatically after exposure to sulfamethoxazole. Sulfamethoxazole treatment resulted in a decrease in the community diversity, while increasing the abundance of pathogenic bacteria. The proportion of *Vibrio* species have increased greatly after treated by sulfamethoxazole, caused *S. variegatus* to be more susceptible to diseases, because the *Vibrio* species are the main opportunistic bacterial pathogens responsible for diseases in marine animals. Besides, the effect of lower concentration of sulfamethoxazole treatment on microbial diversity was greater than that of higher concentration treatment. Our findings provide preliminary guidance for the use of sulfamethoxazole in *S. variegatus*, and lay the foundation for the development of appropriate treatment regimens to address the impact on the gut microbiota. However, deeper research should be continued to reveal the effects of different sulfamethoxazole concentrations on particular types of microbes in the *S. variegatus* gut and thus evaluate their specific functional and molecular contributions to the gut microbiota.

## Data availability statement

The datasets presented in this study can be found in online repositories. The names of the repository/repositories and accession number(s) can be found at: https://www.ncbi.nlm.nih.gov/, PRJNA874578.

## Author contributions

CT and GY conceived and designed the study. CT, WZ, and WW performed sampling and laboratory testing. CT, ZM, and XC analyzed the data and wrote the manuscript. All authors contributed to the article and approved the submitted version.

## Funding

This work was supported by the Earmarked Fund for Hainan Natural Science Foundation for Youth (319QN341), Modern Agro-industry Technology Research System (CARS-49), and National Key R&D Program of China (2019YFD0900905).

## Conflict of interest

The authors declare that the research was conducted in the absence of any commercial or financial relationships that could be construed as a potential conflict of interest.

## Publisher’s note

All claims expressed in this article are solely those of the authors and do not necessarily represent those of their affiliated organizations, or those of the publisher, the editors and the reviewers. Any product that may be evaluated in this article, or claim that may be made by its manufacturer, is not guaranteed or endorsed by the publisher.
